# Application of Antibody-Mediated Therapy for Treatment and Prevention of *Clostridium difficile* Infection

**DOI:** 10.3389/fmicb.2018.01382

**Published:** 2018-06-25

**Authors:** Beatrix Förster, Pui Khi Chung, Monique J. T. Crobach, Ed J. Kuijper

**Affiliations:** ^1^Department of Medical Microbiology, University Medical Center Utrecht, Utrecht, Netherlands; ^2^Bijvoet Center for Biomolecular Research, Utrecht University, Utrecht, Netherlands; ^3^Department of Medical Microbiology, Centre for Infectious Diseases, Leiden University Medical Center, Leiden, Netherlands

**Keywords:** *Clostridium difficile*, antibody, therapy, systemic, oral

## Abstract

*Clostridium difficile* causes antibiotic- and healthcare-associated diarrhea, which is characterized by a high mortality rate (5–15%) and high recurrence rate of 20% or more. Therapeutic alternatives to antibiotics are urgently needed to improve the overall cure rate. Among these, therapeutic antibodies have shown promising results in clinical studies. Herein, the authors review current monoclonal and polyclonal anti- *C. difficile* antibodies that have entered the clinical development stage, either for systemic administration or by the oral route. The antibodies can be applied as monotherapy or in combination with standard-of-care to treat an infection with *C. difficile* or to protect from a recurrence. Bezlotoxumab is the first antibody for secondary prevention of recurrence of *C. difficile* infection approved by the regulatory agencies in US and Europe. The human monoclonal antibody is administered systemically to patients receiving oral standard-of–care antibiotics. Other antibodies are currently in the clinical pipeline, and some are intended for oral use. They show a good safety profile, high efficacy and low production costs, and can be considered promising therapies of the future. The most promising orally administered drug candidate is a bovine antibody from hyperimmune colostral milk, which is in an advanced clinical development stage. Which antibody will enter the market is dependent on its bioavailability at the site of infection as well as its activity against *C. difficile* toxins, protection against colonization and possible action on spore formation. The antibody must demonstrate a clear benefit in comparison with other available treatment options to be considered for use by clinicians.

## Introduction

*Clostridium difficile* (CD) is an anaerobic spore-forming bacterium that can be present in the gut of asymptomatic CD carriers (Crobach et al., 2018). However, it is also the most important cause of infectious antibiotic- and healthcare-associated diarrhea (Rupnik et al., 2009; Magill et al., 2014). In patients developing symptomatic *C. difficile* infection (CDI), symptoms range from mild diarrhea to fulminant life-threatening colitis (Smits et al., 2016).

The conventional treatment of CDI consists of vancomycin or metronidazole, which are non-selective and lead to further disruption of normal gut microbiota, consequently placing patients at risk for relapse or recurrence. Approximately 25% of CDI patients experience recurrent disease, which is often difficult to treat (Cornely et al., 2012). Alternatively, fidaxomicin is considered to be a specific anti-*C. difficile* antibiotic, but its application is restricted due to high costs. Recently, antibody-mediated therapies for treatment or prevention of CDI have gained attention as alternatives to antibiotic treatment. Here, we discuss the rationale behind antibody-mediated therapy and describe the diverse antibody-mediated therapies (excluding active immunization by vaccines) that are currently studied in the clinic for treatment or prevention (of recurrences) of CDI in humans.

## Role of humoral activity against *C. difficile*

Several studies have demonstrated that the adaptive immune response plays an important role in CDI susceptibility, disease course and risk of recurrence. CDI symptoms are caused by the actions of two large enterotoxins, toxin A (TcdA) and B (TcdB). After the secreted toxins have bound and entered the colonic epithelium, TcdA and TcdB can induce an inflammatory response characterized by chemokine and cytokine production, neutrophil influx, disruption of tight junctions, fluid secretion and cell death (Péchiné and Collignon, 2016; Smits et al., 2016). Some strains, including the more virulent ribotypes 027 and 078, also produce a third toxin, termed binary toxin or CD transferase (CDT). CDT is suggested to cause microtubule-based protrusions on epithelial cells, thereby increasing the adherence of CD to target cells (Smits et al., 2016; Aktories et al., 2018). However, its definite role in pathogenesis remains unclear. CD surface proteins, which mainly act as adhesins, include S-layer proteins, flagellar proteins FliC and FliD and cell wall proteins (Cwps) such as Cwp84. These surface proteins also contribute to the initiation of the inflammatory response via cytokine production and interaction with toll-like receptors (Péchiné and Collignon, 2016). Inflammation induced by the innate immune response promotes an adaptive immune response with memory.

Both antibodies to CD toxins and surface proteins have been described, and the adequacy of the humoral immune response can influence susceptibility to CD colonization and/or progression to CDI. In 55 CDI patients, significantly higher serum IgM anti-SLP (S-layer proteins) antibody levels were found at the initial episode in non-CDI recurring patients compared to patients who went on to develop recurrent CDI (rCDI) (Drudy et al., 2004). Furthermore, serum antibody levels for surface proteins, including FliD and FliC, were significantly higher in control patients than in CDI patients (Péchiné et al., 2005). Therefore, antibodies against CD surface proteins are thought to have a protective role against (re)colonization.

Anti-TcdA and anti-TcdB antibodies can protect colonized patients from progression to CDI or recurrent disease. Anti-toxin antibodies are present in the general population, and antitoxin seropositivity prevalence in the general population of 24 or 66% have been previously reported (Viscidi et al., 1983; Bacon and Fekety, 1994). Anti-TcdA antibody levels have also been studied in hospitalized patients receiving antibiotic treatment. After intestinal colonization with *C. difficile*, greater increases in serum levels of anti-TcdA IgG were reported in those patients who remained asymptomatic, compared to those who progressed to CDI. Serum anti-TcdA IgG increased rapidly after colonization, indicating the presence of a systemic anamnestic response to TcdA after day three (Kyne et al., 2000). Furthermore, the development of higher serum anti-TcdB and anti-TcdA IgG levels and fecal anti-TcdA IgA levels during the course of CDI were all reported to be associated with a lower risk of rCDI (Aronsson et al., 1985; Warny et al., 1994). In another study, higher anti-TcdA IgM and IgG levels at day 12 were associated with a lower risk of recurrence (Kyne et al., 2001). Anti-toxin antibodies may be directly protective against disease by neutralizing secreted toxin. The association of anti-toxin antibodies with decreased recurrence risk may be explained by their ability to remove the toxins and stabilize the gut epithelium and microbiota (Dieterle and Young, 2017).

## Antibodies for systemic administration to prevent (relapsing) CDI

### Actoxumab and bezlotoxumab

In 2017, bezlotoxumab (Zinplava©) was approved by the Food and Drug Administration (FDA), and 1 year later by the European Medicines Agency (EMA), as the first monoclonal antibody for prevention of rCDI in patients ≥18 years old.

Actoxumab and bezlotoxumab were developed in parallel as fully human monoclonal IgG (HuMAbs) directed against TcdA and TcdB respectively. Inactivated TcdA and TcdB (both from Techlab), and additionally recombinant TcdB, were used as antigens to immunize mice transgenic for human immunoglobulin genes (HuMAb mice, Medarex Inc., Bloomsbury, New Jersey) (Babcock et al., 2006). Various hybridomas were obtained and screened *in vitro* and *in vivo*. The lead candidate against TcdA (CDA1) recognized the C-terminal region of TcdA and could prevent binding to a cognate receptor on the surface of target cells. The lead candidate against TcdB (MDX-1388) was proposed to bind to the N-terminal domain (putative cell-binding region) of TcdB (Table 1).

**Table 1:** Clinically tested antibody-mediated therapeutic agents for CDI.

**Agent**	**Manufacturer**	**Type of antibody**	**Mode of action**	**State of development**	**Indication**
**SYSTEMIC APPLICATION ROUTE**					
Actoxumab	Merck Sharp & Dohme Ltd (Europe), Inc.	Antitoxin A (CDA1, MK-3415) human monoclonal IgG antibody	Prevents binding to a cognate receptor	Terminated after phase III trial	None
Bezlotoxumab	Merck Sharp & Dohme Ltd (Europe), Inc.	Antitoxin B (CDB1, MDX-1388, MK-6072) human monoclonal IgG antibody	Prevents binding to a cognate receptor	FDA and EMA approved	rCDI
IVIG	Various	Human IgG	Neutralization of TcdA/TcdB	Market (for other indications)	rCDI/CDI/severe CDI
**ORAL APPLICATION ROUTE**					
IgAbulin	ImmunoAG Vienna/Baxter	Human IgA	Neutralization of TcdA/TcdB	Market (for other indications)	CDI
Oral hyperimmune bovine immunoglobulin concentrate (BIC)	Beth Israel Deaconess Medical Center	Polyclonal antibody-enriched colostral immune whey protein concentrate	Neutralization of TcdA/TcdB	Clinical phase I	CDI
MucoMilk	MucoVax BV	Polyclonal antibody-enriched milk immune whey protein concentrate	Neutralization of TcdA/TcdB, Binding of vegetative bacterial cells	Clinical development	rCDI
Cediff	Novatreat Ltd.	Polyclonal antibody-enriched colostral immune whey protein concentrate	Binding of toxigenic vegetative bacterial cells	Interrupted in clinical phase II	rCDI
IMM-529	Immuron Ltd.	Immune colostrum concentrate	Neutralization of TcdB, Binding of vegetative bacterial cells and endospores	Clinical phase I/II	CDI/rCDI

*IVIG, intravenous immunoglobulin; TcdA, C. difficile toxin A; TcdB, C. difficile toxin B; CDI, C. difficile disease; rCDI, recurrent CDI*.

A combination treatment with CDA1 and MDX-1388 in a hamster model for primary disease and relapse showed the best outcome. After safety and pharmacokinetics of CDA1 were assessed in healthy volunteers in a phase I clinical trial (Taylor et al., 2008), efficacy of combination treatment with CDA1 and MDX-1388 was examined in a randomized double-blind, placebo-controlled phase II clinical trial (Lowy et al., 2010). The addition of both antibodies to either metronidazole- or vancomycin-treated patients with CDI significantly reduced recurrence rates. The recurrence rate of patients in the treatment group was 7%, compared to 25% in the placebo group (Table 2). No significant differences were found in disease severity, number of days to resolution, or proportion of treatment failure. Adverse events were reported to be similar in both groups.

**Table 2:** Studies evaluating antibody-mediated therapeutics in humans.

**Agent**	**Study (author, year)**	**Study design**	**Efficacy**
**SYSTEMIC APPLICATION ROUTE**			
Actoxumab (CDA1)	Wilcox et al., 2017	*Clinical Phase III study* MODIFY I:	
		Addition of actoxumab, bezlotoxumab, a-b or placebo to SoC treatment in adult (r)CDI patients	Higher mortality and more AE in actoxumab group; evaluation terminated
Bezlotoxumab (MDX1388)	Wilcox et al., 2017	*Clinical Phase III study* Addition of bezlotoxumab (*n* = 781) vs. placebo (*n* = 773) to SoC treatment in adult (r)CDI patients	
		MODIFY I	No difference initial cure rate (77% vs. 83%) Significant lower rCDI (17% vs. 28%)
		MODIFY II	No difference initial cure rate (83% vs. 78%) Significant lower rCDI (16% vs. 26%)
Actoxumab-bezlotoxumab	Lowy et al., 2010	*Clinical Phase II study*Addition of CDA1+ and MDX1388 in single infusion (*n* = 101) vs. placebo (*n* = 99) to SoC treatment for CDI	Significant lower laboratory-documented rCDI: 7% comparator vs. 25% placebo
	Wilcox et al., 2017	*Clinical phase III study* Addition of actoxumab-bezlotoxumab (*n* = 773) vs. placebo (*n* = 773) to SoC treatment in adult (r)CDI patients	
		MODIFY I	No difference initial cure rate (75% vs. 83%) Significant lower rCDI(16% vs. 28%)
		MODIFY II	No difference initial cure rate (72% vs. 78%) Significant lower rCDI (15% vs. 26%)
IVIG	Leung et al., 1991	*Case series*5 pediatric patients with hypoglobulinaemia and rCDI, 400 mg/kg IVIG	All patients had full resolution of symptoms
	Wilcox, 2004; McPherson et al., 2006; Negm et al., 2017	*Retrospective studies* 36 adult patients with (r)CDI, 150–500 mg/kg IVIG	24 patients showed therapeutic response, 12 did not respond
	Juang et al., 2007; Shahani and Koirala, 2015	*Retrospective studies* 39 adult patients with severe CDI, 82 control patients, 400 mg/kg IVIG	No significant differences regarding outcome and severity of symptoms
**ORAL APPLICATION ROUTE**			
IgAbulin	Tjellström et al., 1993	*Case study* 1 pediatric patient with severe CDI	Full resolution of symptoms
MucoMilk	van Dissel et al., 2005	*Prospective cohort study* 15 adult patients, 1 pediatric patient with completed antibiotic therapy, under which 9 patients with rCDI	No relapse within a median follow-up period of 333 days
	Numan et al., 2007	*Prospective cohort study* 101 adult patients, with completed antibiotic therapy, 61 patients with CDI 40 patients with rCDI (40%)	Prevention of relapse by 50% during a follow up period of 60 days
Cediff	Mattila et al., 2008	*Prospective study* 38 adult patients with rCDI 20 were treated with Cediff, 18 were treated with metronidazole	Cediff was as effective as metronidazole in the prevention of CDI recurrences during a 70 day follow up (sustained recovery 56% vs. 55%)

*a-b, actoxumab-bezlotoxumab; AE, adverse event; SOC, standard-of-care; CDI, C. difficile disease; CDA1, human monoclonal anti-toxin A; CDB1, human monoclonal anti-toxin B; rCDI, recurrent CDI; SoC, standard-of-care antibiotic treatment for CDI; TcdA, C. difficile toxin A; TcdB, C. difficile toxin B; vs., versus; IVIG, intravenous immunoglobulin*.

The worldwide development and commercialization rights for both antibodies were purchased by Merck for US$ 60 million, (Markham, 2016) and two double-blind, placebo-controlled phase III trials, MODIFY I and II, were conducted to assess efficacy and safety of both antibodies (subsequently named actoxumab and bezlotoxumab) (Wilcox et al., 2017). MODIFY I had an adaptive design, allowing enrolment cessation in any group after interim analysis. Patients were randomly assigned in a 1:1:1:1 ratio to either receive a single dose of bezlotoxumab, actoxumab, bezlotoxumab plus actoxumab or placebo in addition to standard antibiotic treatment for CDI. During the interim analysis of MODIFY I, higher mortality and more serious adverse events were found in the actoxumab group, causing enrolment in this group to be terminated. Subsequently, actoxumab was not evaluated in MODIFY II. While in both trials initial cure rates did not differ between antibody-treated groups and placebo groups, significantly lower recurrence of CDI was found in the bezlotoxumab groups during 12 weeks post-treatment, as recurrent infections were seen in 17 and 16% in these groups in MODIFY I and II respectively, in comparison to 28 and 26% in the placebo groups (Table 2). Subgroup analysis of initially cured participants similarly showed significant differences in the recurrence rate, while sensitivity analysis taking into account missing data and early discontinuations were consistent with these findings. The addition of actoxumab to bezlotoxumab did not improve efficacy in MODIFY I. The overall safety profile of bezlotoxumab was favorable, though the assessment reports of FDA and EMA noted a numerical increment of heart failure and all-cause mortality among bezlotoxumab treated subjects with baseline congestive heart failure, compared to placebo-treated patients.

The benefit of bezlotoxumab and actoxumab-bezlotoxumab was confirmed in subpopulations at high risk for rCDI or CDI complications. *Post-hoc* analyses of the two phase 3 trials (MODIFY 1 and MODIFY II) showed greater benefit of bezlotoxumab than placebo in patients with at least three risk factors. No difference in CDI recurrence was observed for patients with no predefined risk factors (Gerding et al., 2018).

Bezlotoxumab is available for the retail price of $4,560 per vial (Lee et al., 2017). In a model-based analysis of the MODIFY trial subjects by the manufacturer, bezlotoxumab was reported to be cost-effective compared to placebo (Prabhu et al., 2018), although no reports of comparison to other approaches such as fecal transplant or fidaxomicin treatment have been published.

### Intravenous immunoglobulin

In contrast to monoclonal antibody therapies, a cocktail of synergistic polyclonal antibodies has the ability to bind multiple targets and mediate a variety of effector functions (Wang et al., 2013).

Intravenous immunoglobulin (IVIG) is in use for numerous clinically approved pathological conditions (Hässig, 1967; Stiehm et al., 2008), and in more than 100 different indications as an off-label drug (João et al., 2018). The rationale to apply IVIG to patients with CDI is based on the prevalence of serum antibodies against TcdA and TcdB in healthy blood donors (Viscidi et al., 1983; Bacon and Fekety, 1994), and a low antitoxin serum level in patients that are predisposed for recurrent, prolonged or severe CDI (Salcedo et al., 1997; Kyne et al., 2000; Table 1). After a successful study in children (Leung et al., 1991) IVIG has been used in several small, mostly retrospective and non-randomized studies with elderly patients suffering from recurrent (Beales, 2002; Wilcox, 2004) or severe CDI (Salcedo et al., 1997; McPherson et al., 2006; Juang et al., 2007; Shah et al., 2014; Shahani and Koirala, 2015; Table 2). So far, there is a lack of robust evidence of a beneficial therapeutic effect of IVIG due to the absence of a sufficiently powered randomized controlled trial (Negm et al., 2017). In 2005, a randomized placebo- controlled interventional study planned for 40 patients with severe relapsing or refractory CDI (ClinicalTrials.gov NCT00177970) was started and prematurely terminated. The sponsor states that they were unable to receive IVIG free of cost from a pharmaceutical company to continue.

In addition to the lack of consensus regarding optimal dose and timing of IVIG administration and patient selection, the quality of IVIG is a pivotal factor. Plasma or blood of 1,000–100,000 healthy donors is used for IVIG manufacturing and the compositions of plasma donor pools are not standardized. Recently, three commercially available IVIG preparations were screened for their reactivity to a panel of seven CD antigens. They showed significant differences in antigen binding and toxin neutralization, which are considered as the predominant mode of action of IVIG against CDI (Negm et al., 2017). Selected sourcing of donors with naturally occurring anti-toxin antibodies for the manufacturing of IVIG with standardized elevated specific antibody levels is feasible (Wasserman et al., 2016). It is questionable if the market size of severe and rCDI and predominance of low-cost medication can offer an incentive to invest into a specialized IVIG.

As a blood-derived product, IgG pool carries a potential (low) risk for blood-borne disease transmission (Chapel, 1999) and contains batch-to-batch variability. Additionally, only a small fraction of antibodies bind the target of interest. The biodistribution of IVIG outside the bloodstream is poorly understood (Brandtzaeg and Baklien, 1976; Wasserman et al., 2012; Nikpoor et al., 2015), and it is unclear if the concentration of antibody that reaches the site of infection could be sufficient to exert the desired effect.

Furthermore, IVIG may not be cost-effective and would not relieve the financial burden on health care. While each CDI episode costs $3,500–5,042 (Dubberke et al., 2008), each IVIG administration is calculated at $4551.25 (Blackhouse et al., 2010).

## Antibodies for oral application against CDI

Oral delivery of antibodies to target pathogens restricted to the gastrointestinal tract provides a highly attractive treatment strategy. A needle-free application directly on the affected site may result in a higher efficacy with reduced side-effects at a lower dose (Jones and Martino, 2016). Human and bovine antibodies have been used in the past to treat CDI.

### Human antibodies

A human IgA and IgG pool (Tjellström et al., 1993; Saturno, 2006) derived from healthy blood donors was successfully used to orally treat children with severe CDI resistant to standard treatment (Table 2). Consistent with findings for infants orally treated with human IgG (Blum et al., 1981), IgA remained restricted to the gastrointestinal tract and did not enter the blood stream (Casswall et al., 1996).

### Bovine antibodies

Hyperimmune bovine colostrum (HBC) or milk is an easily scalable and cost-effective source (USD$1/gram antibody; Hutton et al., 2017) for orally applicable antibodies with a strong safety profile. It is produced by vaccination of cows during gestation, and is rich in targeted IgG or secretory IgA (sIgA).

HBC derived from cows that were immunized with recombinant mutants of TcdA and TcdB showed a therapeutic efficacy in gnotobiotic piglets with diarrhea due to CDI (Sponseller et al., 2015).

A single-site, open, crossover, clinical phase I study on six healthy volunteers was performed to analyse the survival of bovine IgG in the human gastrointestinal tract after application of a single oral dose. Antibody-enriched colostral immune whey protein concentrate (BIC) was derived from cows immunized with either a toxoid derived from culture medium of toxigenic CD containing both TcdA and B, or a toxoid of purified TcdA (Kelly et al., 1997). Antibody within enteric coated capsules could be recovered to about 30% of the initial dose in stool while retaining its specific toxin-neutralizing activity. Based on *in vitro* and preclinical findings, it was assumed that the bovine IgG could neutralize CD toxins within the colonic lumen (Kelly et al., 1997). This study also claimed bovine IgG concentrate against CD to be safe for oral use in humans. The following single site, open, phase I study was performed on six volunteers with an end ileostomy to examine safety and bioavailability of a single oral dose. This study showed the recovery of about 50% of the orally applied antibody and a retained activity. It also revealed the difficulty of properly formulating the oral antibody therapy for patients with abnormal mouth to ileum transit times (Warny et al., 1999).

Immune whey protein concentrate derived from mature milk of cows vaccinated with formaldehyde-inactivated whole CD cells (VPI10463) and toxoid prepared from the CD culture filtrate contained a high concentration of specific sIgA antibodies (Schmautz et al., 2018; Table 1). This product, named MucoMilk, was intended to serve as “clinical nutrition” (not medication) for patients with CDI and considered to be safe (Young et al., 2007). The prevention of relapses of CDI was evaluated in a prospective uncontrolled cohort study enrolling 16 patients with CDI. The whey concentrate was applied subsequently to standard antibiotic treatment. During a follow-up period of median 333 days, none of the patients had experienced a relapse (van Dissel et al., 2005). A following non-blinded, clinical cohort study applying the same treatment scheme on 101 patients showed a reduction of recurrence by about 50% (van Dissel et al., 2005; Numan et al., 2007; Bauer et al., 2008; Bauer and van Dissel, 2009; Table 2).

Next, a controlled, double-blind, randomized, parallel-group, multicenter trial with concentrated colostral immune whey from cows vaccinated with formaldehyde-inactivated whole CD cells (T-37067, T-36842), named Cediff, was performed (Table 1). Cediff was compared with metronidazole against rCDI in patients that already encountered at least one episode. Although only a small number of patients were included, the recurrence rate of both treatment groups was similar (Mattila et al., 2008; Table 2). Further development was stopped.

Recently, a phase 1/2, randomized, double blind, placebo-controlled clinical study started enrolling patients with primary and rCDI, to evaluate safety and tolerability of hyper-immune bovine colostrum powder (IMM-529) together with standard-of-care (ClinicalTrials.gov NCT03065374). IMM-529 is derived from cows vaccinated with TcdB, vegetative cells and endospores of CD (Hutton et al., 2017; Table 1). In contrast to previous approaches (Korhonen et al., 2000) the immune colostrum powder is produced as pharmaceutical.

## Perspective

Further antibodies against CDI are in preclinical development, such as avian antibodies against TcdA and B (Kink and Williams, 1998) or spores (Pizarro-Guajardo et al., 2017) and fully human polyclonal IgG obtained by immunization of transgenic cattle with a trivalent and quadravalent toxin vaccine (Tian et al., 2017).

Smaller antibody formats such as a tetravalent bispecific heavy-chain-only single domain (VHH) antibody against TcdA and B features neutralization in a subnanomolar range and efficacy in animal models of CDI and is deliverable by gene therapy (Yang et al., 2014, 2016). Engineered lactobacilli that produce variable domains of heavy chain only antibodies against CD toxins show partial protection in a hamster model (Andersen et al., 2016).

Fully functional antibodies that consist of heavy chains only are named nanobodies. Nanobodies against binary toxin (Unger et al., 2015) and surface proteins of CD (Kandalaft et al., 2015) as well as variable domains derived from cartilaginous shark IgNAR (Krah et al., 2016) have shown effects *in vitro*. A camelid single domain antibody against TcdA could be easily optimized by an affinity maturation platform and could serve as an alternative therapeutic modality in the future (Sulea et al., 2018). A novel humanized monoclonal antibody recognizes a single, highly conserved epitope on the TcdB glucosyltransferase domain and neutralizes TcdB from various CD strains (Kroh et al., 2018).

## Conclusion

Taken together, antibodies such as bezlotoxumab have proven to be capable of protecting from rCDI, while mostly being directed against toxins. A better understanding of virulence factors of CD could help to broaden the repertoire of therapeutic targets and may result in antibodies also applicable for severe and refractory CDI or CDI caused by “hypervirulent” strains. Due to their property of maintaining the integrity of the gut microflora and providing no selective pressure for escape mutants (Steele et al., 2013), polyclonal antibodies could act synergistically with antibiotics and FMT and could easily be integrated in treatment regimens, provided that their costs are competitive with current therapies. Oral antibodies have the clear advantage that they could be applied not only at the clinic but also at the patient's home.

## Author contributions

BF, PC, MC, and EK have written the manuscript and compiled the tables.

### Conflict of interest statement

The authors declare that the research was conducted in the absence of any commercial or financial relationships that could be construed as a potential conflict of interest.
